# New Onset and Exacerbation of Psoriasis Following COVID-19 Vaccination: A Review of the Current Knowledge

**DOI:** 10.3390/biomedicines11082191

**Published:** 2023-08-03

**Authors:** Luca Potestio, Teresa Battista, Sara Cacciapuoti, Angelo Ruggiero, Fabrizio Martora, Luigi Fornaro, Elisa Camela, Matteo Megna

**Affiliations:** 1 Section of Dermatology, Department of Clinical Medicine and Surgery, University of Naples Federico II, Via Pansini 5, 80131 Napoli, Italy; 2 Dermatology Unit, Istituto Dermopatico dell’Immacolata—IRCCS, 00144 Rome, Italy

**Keywords:** psoriasis, COVID-19 vaccination, psoriasis exacerbation, psoriasis flare, de novo psoriasis, new-onset psoriasis

## Abstract

COVID-19 vaccination was the main measure to overcome the pandemic. As with other drugs and vaccines, mild to moderate adverse events have been reported following vaccination. In addition, several cutaneous reactions have been described. In particular, there are several reports investigating de novo psoriasis or the exacerbation of psoriasis following COVID-19 vaccination. However, data on the possible pathogenetic mechanisms as well as comprehensive manuscripts on the topic are scant. Thus, the aim of our manuscript was to perform a review of the current literature on post-COVID-19 vaccination exacerbations and new-onset psoriasis in order to offer a wide perspective on this area and to point out possible pathogenetic mechanisms. Research on the current literature was performed following PRISMA guidelines. In total, 49 studies involving 134 patients developing new-onset psoriasis (*n* = 27, 20.1%) or psoriasis exacerbation (*n* = 107, 79.9%) were collected. Although cases of de novo psoriasis or a worsening of psoriasis have been reported following vaccination, all of the cases have been successfully treated while overall benefit–risk profile of COVID-19 vaccination does not justify vaccine hesitancy due to the risk of psoriasis being developed or worsening. Certainly, further studies are needed to identify possible pathogenetic mechanisms in order to identify “at-risk” patients. Finally, vaccination should not be discouraged.

## 1. Introduction

Psoriasis is a chronic, inflammatory skin disorder that affects millions of individuals worldwide (with up to a 3% prevalence) [1,2]. Clinically, it is characterized by the presence of thick, red, scaly patches on the skin’s surface [2,3]. Moreover, several comorbidities can be associated with the psoriatic disorder (hypertension, dyslipidemia, obesity, psoriatic arthritis, anxiety/depression, inflammatory bowel disease, diabetes mellitus, etc.), making this disease a burden for patients’ mental and emotional well-being, leading to social isolation and a reduction in quality of life [4,5,6]. Therefore, psoriasis treatment is not limited to skin lesions but also to its comorbidities and the psychosocial aspects of the disease [7,8,9]. Currently, several treatment options for psoriasis are available. These include topical treatments (creams and ointments) which may be used for the mild form of the disease, phototherapy (exposure to ultraviolet light), conventional systemic medications (cyclosporin, methotrexate, acitretin, and fumarates), small molecules, and biologic therapies which are used for moderate-to-severe forms [10,11,12,13]. In particular, the introduction of biologic drugs specifically targeting interleukins (IL) 23 and 17 and tumor necrosis factor-alpha (TNFα), involved in psoriasis pathogenesis, revolutionized the management of the disease, showing promising results in terms of effectiveness and safety [14,15,16]. Globally, treatment plans are tailored to everyone’s specific needs, taking into account the severity of the disease, its impact on a patient’s quality of life, and the comorbidities [17,18].

The COVID-19 pandemic, caused by the novel coronavirus SARS-CoV-2, has had a profound impact on global health, economies, and societies worldwide [19,20]. Due to the high transmissibility of the virus, preventive measures such as wearing masks, practicing physical distancing, and frequent hand hygiene played a crucial role in mitigating transmission [21,22]. As regards dermatological clinical practice, teledermatology emerged as a valuable tool in providing remote dermatological care during the pandemic. Indeed, it has played a vital role in maintaining access to dermatological care, reducing viral transmission, improving access to care, and ensuring the continuity of treatment [23,24]. Globally, the introduction of COVID-19 vaccination was the main measure to overcome the pandemic. Indeed, COVID-19 vaccination played a vital role in controlling the spread of the virus and reducing the severity of the disease [25,26,27]. Multiple vaccines have been developed and authorized for emergency use around the world, working by stimulating the immune system to recognize and respond to the SARS-CoV-2 virus, preventing infection or reducing the severity of illness if infection occurs [25,26,27]. In particular, four vaccines have been approved by the European Medicines Agency (EMA), based on two different mechanisms of action: mRNA-based vaccines (Pfizer/BioNTech;BNT162b2 and Moderna; mRNA-1273) and viral vector-based vaccines (AstraZeneca; AZD1222 and Johnson & Johnson; Ad26.COV2.S) [25,26,27].

As with other drugs and vaccines, mild to moderate adverse events (AEs) have been reported following vaccination, including fatigue, diarrhea, headache, fever, muscle aches, pain or redness at the injection site, chills, etc. [28,29]. Fortunately, most of these reactions have been mild and self-limited. In addition, several cutaneous reactions have been described following COVID-19 vaccination [28,29]. In particular, there are several reports investigating de novo psoriasis or an exacerbation of psoriasis following COVID-19 vaccination [30,31]. However, data on the possible pathogenetic mechanisms as well as comprehensive manuscripts on the topic are scant. Thus, the aim of our manuscript was to perform a review of the current literature on post-COVID-19 vaccination exacerbations and new-onset psoriasis in order to offer a wide perspective on COVID-19 vaccination and psoriasis and to point out possible pathogenetic mechanisms.

## 2. Materials and Methods

Research on the current literature was performed using the following databases: PubMed, Cochrane Skin, Embase, EBSCO, MEDLINE, and Google Scholar (up to 1 June 2023). Studies were identified, screened and extracted for relevant data following PRISMA (preferred reporting items for systematic reviews and meta-analyses) guidelines [32], using the following keywords: “COVID-19”, “vaccine”, “cutaneous”, “vaccination”, “side effects”, “adverse events”, “safety”, “efficacy”, “skin manifestations”, “mRNA”, “viral vector”, “Pfizer/BioNTech”, “BNT162b2”, “Moderna”, “mRNA-1273”, “AstraZeneca”, “Johnson & Johnson”, “Ad26.COV2.S”, “AZD1222”, and “psoriasis”. The manuscripts analyzed included reviews, meta-analyses, letters to the editor, real-world studies, and case series. Manuscripts that fit the aim of our review were considered. Studies reporting at least one patient who developed new-onset psoriasis or experienced a worsening of psoriasis following at least one dose of COVID-19 vaccine were included. Only the BNT162b2, mRNA-1273, AZD1222 and Ad26.COV2.S vaccines were considered in our review. Studies reporting de novo psoriasis or an exacerbation of psoriasis following other types of vaccines were excluded and studies investigating psoriatic arthritis were not considered. All the clinical phenotypes of psoriasis were included (plaque, guttate, pustular, erythrodermic, inverse, palmoplantar, etc.). The search was then refined by reviewing the texts and abstracts of the collected articles. The bibliography was also revised to include articles that may have been missed. Only English-language manuscripts were considered.

This article is based on previously conducted studies and does not contain studies with human or animal participants conducted by any of the authors.

## 3. Results

**Figure 1:**
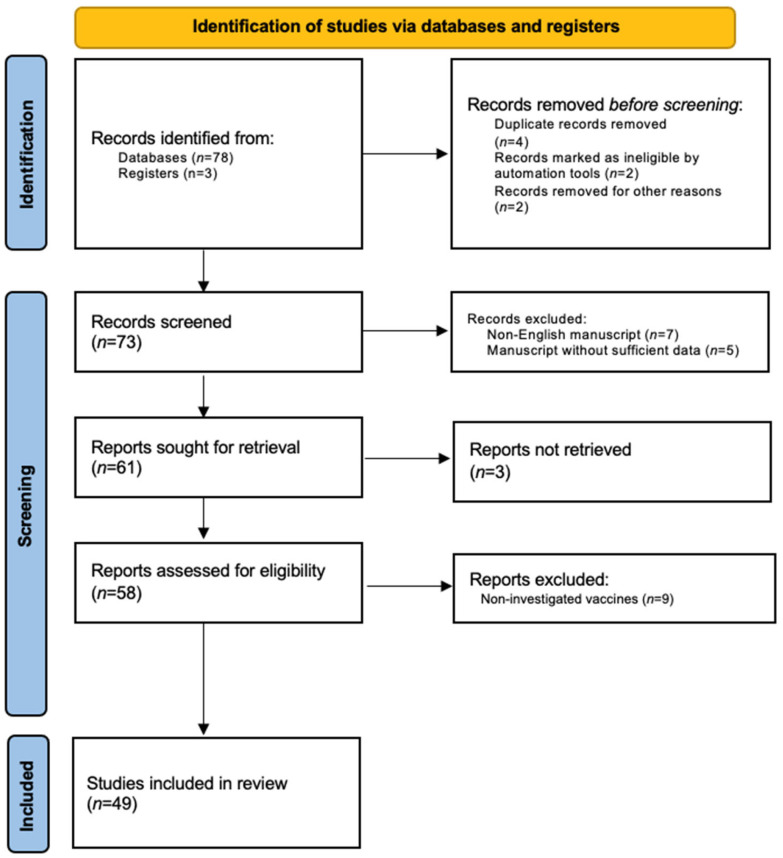
PRISMA flow-chart.

In total, 49 studies involving 134 patients were included in this review (Figure 1) and are summarized in Table 1 (new-onset psoriasis) [28,29,30,31,32,33,34,35,36,37,38,39,40,41,42,43,44,45,46,47] and Table 2 (psoriasis exacerbation) [32,33,34,35,39,45,48,49,50,51,52,53,54,55,56,57,58,59,60,61,62,63,64,65,66,67,68,69,70,71].

### 3.1. New-Onset Psoriasis

New-onset psoriasis refers to the development of psoriasis in individuals who previously did not have the condition. The exact triggers for new-onset psoriasis can vary among individuals, but potential factors include infections, physical trauma to the skin, stress, certain medications, and hormonal changes. As regards cases of new-onset psoriasis following COVID-19 vaccination, a total of 27 (male: 10 (37.0%); female: 13 (48.1%); not reported: 4 (14.8%); mean age: 54.4 ± 20.9 years) cases reported in 20 manuscripts are described and summarized in Table 1 [28,29,30,31,32,33,34,35,36,37,38,39,40,41,42,43,44,45,46,47]. In particular, plaque psoriasis was the commonest clinical phenotype (*n* = 9 (33.3%)), followed by guttate (7 (25.9%)), pustular (4 (14.8%)), nail (3 (11.1%)) and annular psoriasis (1 (3.7%)), while the clinical phenotype of the remaining 3 (11.1%) subjects has not been reported. Moreover, mRNABNT162b2 was the commonest vaccine associated with psoriasis development (*n* = 15, 55.5%), followed by AZD1222 (*n* = 5, 18.5%) and mRNA-1273 (*n* = 3, 11.1%). Notably, psoriasis development was reported following all of the doses of vaccination, with some patients experiencing psoriasis disease after each single dose. The mean time between vaccine administration and new-onset psoriasis was 10.3 ± 6.4 days (range: 2–30 days) (only cases with reported data were counted). Finally, the majority of cases of de novo psoriasis were mild. Indeed, topical treatments (a topical calcipotriol/betamethasone combination and topical corticosteroids) were successfully used in nine (33.3%) patients, limiting the use of conventional systemic drugs (oral corticosteroids, acitretin and methotrexate) and biologics to three (11.1%) and five (18.5%) cases, respectively. Of note, therapy was not reported in seven (25.9%) patients whereas the remaining subjects were managed with apremilast (*n* = 2, 7.4%) or without treatment (*n* = 1, 3.7%).

**Table 1:** New-onset psoriasis following COVID-19 vaccination.

Authors	Country	Cases	Age/Sex	Type of Psoriasis	Vaccines/Doses	Days *	After
Tran et al. [33]	Vietnam	3	Pt1: 51/M	Pt1: plaque	Pt1: AZD1222/3	Pt1: 7	Calcip/betam + antihistamines
			Pt2: 68/F	Pt2: plaque	Pt2: BNT162b2/3	Pt2: 30	
			Pt3: 73/M	Pt3: guttate	Pt3: BNT162b2/1	Pt3: 30	
Català A et al. [35]	Spain	3	NR	NR	NR	NR	NR
Nagrani et al. [36]	India	2	Pt1: 56/F	Pt1: plaque	Pt1: AZD1222/1-2	Pt1: 7-2	Apremilast + antihistamines + emollients
			Pt2: 65/M	Pt2: plaque	Pt2: AZD1222/2	Pt2: 10	
Ouni et al. [37]	Tunisia	2	Pt1: 59/M	Pt1: guttate	Pt1: BNT16B2b2/1-2-3	Pt1: 7-7-7	Pt1: TCS
			Pt2: 23/F	Pt2: guttate	Pt2: Ad26.COV2.S/1	Pt2: 2	Pt2: TCS
Gargiulo et al. [34]	Italy	2	Pt1: 82/F	Pt1: plaque	Pt1: BNT162b2/3	NR	Pt1: secukinumab
			Pt2: 29/M	Pt2: pustular	Pt1: BNT162b2/2		Pt2: guselkumab
Cortonesi et al. [38]	Italy	1	82/F	Plaque	BNT162b2/1	7	Ixekizumab
Wei et al. [39]	USA	1	24/M	Plaque	mRNA-1273/2	24	Ixekizumab + acitretin 25 mg/die
Ständer et al. [40]	Germany	1	50/F	Plaque	BNT162b2/2	7	None
McMahon et al. [41]	USA	1	67/NR	Plaque	mRNA-1273/NR	NR	NR
Song et al. [42]	Korea	1	23/F	Guttate	BNT162b2/1	2	Calcip/betam
Magro et al. [43]	USA	1	58/M	Guttate	BNT162b2/2	14	NR
Pesqué et al. [44]	Spain	1	72/M	Guttate	mRNA-1273/2	6	Calcip/betam
Lehmann et al. [45]	Switzerland	1	79/F	Guttate	BNT162b2/1	10	Calcip/betam + nbUVB
Frioui et al. [46]	Tunisia	1	20/M	Pustular	BNT162b2/1	4	Acitretin 25 mg/die + TCS
Elamin et al. [47]	United Kingdom	1	66/F	Pustular	AZD1222/1	21	Acitretin 20 mg/die
Romagnuolo et al. [48]	Italy	1	64/F	Pustular	BNT162b2/1	NR	Methotrexate 15 mg per week
Ricardo et al. [49]	USA	1	76/F	Nail	BNT162b2/2	7	TCS
Ruggiero et al. [50]	Italy	1	47/F	Nail	BNT162b2/2	17	Ixekizumab
Lamberti et al. [51]	Italy	1	45/F	Naile	BNT162b2/2	3	NR
Chhabra et al. [52]	India	1	NR/M	Annular	AZD1222/1	14	NR

M: male; F: female; AZD1222: AstraZeneca-Oxford AZD1222; mRNA-1273: Moderna mRNA-1273; BNT162b2: Pfizer mRNABNT162b2; Ad26.COV2.S: Johnson & Johnson; calcip/betam: topical calcipotriol/betamethasone combination; nbUVB: narrow-band UVB; TCS: topical corticosteroids; OCS: oral corticosteroids; NR: not reported. * Number of days between new-onset psoriasis and vaccination.

### 3.2. Psoriasis Exacerbation

Psoriasis exacerbation refers to a worsening or flare-up of symptoms in individuals who already have psoriasis. During an exacerbation, existing psoriasis lesions may become more inflamed, larger, and more widespread. Additionally, new lesions may develop in previously unaffected areas of the body. The severity and duration of an exacerbation can vary from person to person. Several factors can trigger or contribute to a psoriasis exacerbation, including stress, infections, certain medications, changes in weather or climate, hormonal fluctuations, injury or trauma to the skin, and lifestyle factors such as smoking and excessive alcohol consumption. In this scenario, in total, 107 cases (male: 61 (57.0%); female: 46 (43.0%); mean age: 56.5 ± 13.4 years) of a flare-up of psoriasis collected in 29 articles are described and summarized in Table 2 [32,33,34,35,39,45,48,49,50,51,52,53,54,55,56,57,58,59,60,61,62,63,64,65,66,67,68,69,70,71]. Of these, 74 (69.2%), 12 (11.2%), 8 (7.5%), 5 (4.7%), 4 (3.7%) and 4 (3.7%) subjects developed a flare-up of plaque, guttate, pustular, erythrodermic, nail and palmoplantar psoriasis. In particular, mRNABNT162b2 was the commonest vaccine associated with psoriasis development (*n* = 66, 61.7%), followed by AZD1222 (*n* = 21, 19.6%) and mRNA-1273 (*n* = 20, 18.7%). Of note, psoriasis development was reported following all of the doses of vaccination. The mean time between vaccination and psoriasis worsening was 13.7 ± 14.4 days (range: 2–90 days) (only cases with reported data were counted). Differently from new-onset psoriasis, there are 5 case series reporting at least 10 cases of psoriasis exacerbation following vaccination. Of these, Gargiulo et al. reported the largest cohort of cases (*n* = 16), followed by Huang et al. [53] (*n* = 15) and Sotiriou et al. (*n* = 14) [54]. Finally, even though psoriasis therapy before and after vaccination was not reported in 57 (53.3%) and 22 cases (20.6%), biologic treatment was the commonest drug administered for the management of psoriasis exacerbation (*n* = 39, 36.4%), followed by the addition of topical treatments to current therapies (a topical calcipotriol/betamethasone combination: 12, 11.2%; topical corticosteroids: 11, 10.3%), conventional systemic drugs (cyclosporin, acitretin and methotrexate) (*n* = 8, 7.5%), and phototherapy (*n* = 5, 4.7%). As regards biologic treatments, 33 (30.8%) patients started biologics for the first time (anti-IL23: 18 (54.5%); anti-IL17: 9 (27.3%); anti-IL12/23: 2 (6.1%); anti-TNFα; 4 (12.1%)) whereas current biologic treatment was switched to in 6 (5.6%) subjects. Differently from de novo psoriasis, several cases of moderate-to-severe forms of disease were reported following vaccination (as well as three cases of erythrodermic psoriasis), leading to the use of conventional systemic drugs and biologics in 47 (44.0%) patients. Notably, 19 (17.8%) patients who developed psoriasis exacerbation were under biologic treatment for psoriasis at the moment of the flare-up.

**Table 2:** Psoriasis exacerbation following COVID-19 vaccination.

Authors	Cases	Age/Sex	Type of Psoriasis	Vaccines/Doses	Days *	Treatment **	After ***
Gargiulo et al. [34]	16	Pt1: 40/F	Pt1: plaque	Pt1: BNT162b2/3	NR	NR	Pt1: tildrakizumab
		Pt2: 50/M	Pt2: plaque	Pt2: BNT162b2/2			Pt2: tildrakizumab
		Pt3: 25/M	Pt3: plaque	Pt3: BNT162b2/2			Pt3: ixekizumab
		Pt4: 40/F	Pt4: plaque	Pt4: mRNA-1273/3			Pt4: tildrakizumab
		Pt5: 53/M	Pt5: plaque	Pt5: BNT162b2/2			Pt5: risankizumab
		Pt6: 50/M	Pt6: plaque	Pt6: BNT162b2/2			Pt6: guselkumab
		Pt7: 38/M	Pt7: plaque	Pt7: BNT162b2/2			Pt7: risankizumab
		Pt8: 56/M	Pt8: plaque	Pt8: BNT162b2/1			Pt8: secukinumab
		Pt9: 50/M	Pt9: plaque	Pt9: mRNA-1273/2			Pt9: brodalumab
		Pt10: 78/M	Pt10: plaque	Pt10: BNT162b2/3			Pt10: risankizumab
		Pt11: 67/F	Pt11: plaque	Pt11: BNT162b2/3			Pt11: risankizumab
		Pt12: 57/M	Pt12: guttate	Pt12: BNT162b2/2			Pt12: guselkumab
		Pt13: 53/M	Pt13: plaque	Pt13: BNT162b2/2			Pt13: bimekizumab
		Pt14: 61/M	Pt14: plaque	Pt14: mRNA-1273/2			Pt14: risankizumab
		Pt15: 72/F	Pt15: plaque	Pt15: BNT162b2/2			Pt15: ustekinumab
		Pt16: 76/M	Pt16: plaque	Pt16: BNT162b2/3			Pt16: guselkumab
Huang et al. [53]	15	NR/8M-7F	8 plaque 7 guttate	AZD1222/1 (3) AZD1222/2 (4) AZD1222/1-2 (1) mRNA-1273/1 (1) mRNA-1273/2 (6)	9.3	NR	NR
Sotiriou et al. [54]	14	Pt1: 69/F	Plaque	Pt1: AZD1222/2	Pt1: 8	NR	Pt1: PUVA
		Pt2: 82/F		Pt2: mRNA-1273/2	Pt2: 10		Pt2: calcip/betam
		Pt3: 62/F		Pt3: BNT162b2/2	Pt3: 6		Pt3: calcip/betam
		Pt4: 73/M		Pt4: BNT162b2/2	Pt4: 7		Pt4: calcip/betam
		Pt5: 66/M		Pt5: AZD1222/1	Pt5: 22		Pt5: risankizumab
		Pt6: 62/F		Pt6: AZD1222/2	Pt6: 13		Pt6: apremilast
		Pt7: 78/F		Pt7: BNT162b2/2	Pt7: 5		Pt7: calcip/betam
		Pt8: 64/F		Pt8: AZD1222/2	Pt8: 6		Pt8: PUVA
		Pt9: 69/M		Pt9: AZD1222/1	Pt9: 32		Pt9: nbUVB
		Pt10: 83/M		Pt10: BNT162b2/2	Pt10: 9		Pt10: calcip/betam
		Pt11: 61/F		Pt11: AZD1222/2	Pt11: 3		Pt11: nbUVB
		Pt12: 49/M		Pt12: BNT162b2/2	Pt12: 10		Pt12: ixekizumab
		Pt13: 55/F		Pt13: BNT162b2/2	Pt13: 7		Pt13: cyclosporine
		Pt14: 64/F		Pt14: AZD1222/2	Pt14: 7		Pt14: guselkumab
Koumaki et al. [55]	12	Pt1: 34/F	Pt1: plaque	Pt1: BNT162b2/2	Pt1: 10	Pt1: secukinumab	Pt1: secukinumab + emollients
		Pt2: 61/M	Pt2: plaque	Pt2: AZD1222/1	Pt2: 14	Pt2: TCS	Pt2: apremilast
		Pt3: 45/F	Pt3: plaque	Pt3: BNT162b2/2	Pt3: 10	Pt3: calcip/betam	Pt3: TCS + calcip/betam
		Pt4: 56/F	Pt4: plaque	Pt4: BNT162b2/2	Pt4: 2	Pt4: adalimumab	Pt4: adalimumab + TCS
		Pt5: 53/F	Pt5: pustular	Pt5: BNT162b2/1	Pt5: 20	Pt5: adalimumab	Pt5: TCS+ OCS
		Pt6: 56/F	Pt6: plaque	Pt6: BNT162b2/1-2	Pt6: 3	Pt6: secukinumab	Pt6: secukinumab
		Pt7: 34/F	Pt7: plaque	Pt7: BNT162b2/1-2	Pt7:7	Pt7: secukinumab	Pt7: secukinumab
		Pt8: 61/F	Pt8: pustular	Pt8: BNT162b2/2	Pt8: 4	Pt8: methotrexate	Pt8: methylprednisolone
		Pt9: 66/F	Pt9: plaque	Pt9: BNT162b2/1	Pt9: 20	Pt9: ustekinumab	Pt9: ustekinumab + TCS
		Pt10: 67/F	Pt10: plaque	Pt10: BNT162b2/2	Pt10: 20	Pt10: OCS	Pt10: TCS + OCS
		Pt11: 56/M	Pt11: plaque	Pt11: BNT162b2/2	Pt11: 20	Pt11: calcip/betam	Pt11: calcip/betam
		Pt12: 51/M	Pt12: plaque	Pt12: BNT162b2/2	Pt12: 25	Pt12: TCS	Pt12: calcip/betam
Megna et al. [56]	11	Pt1: 55/M	Pt1: plaque	Pt1: BNT162b2/2	Pt1: 5	Pt1: none	Pt1: methotrexate
		Pt2: 49/M	Pt2: plaque	Pt2: BNT162b2/2	Pt2: 6	Pt2: none	Pt2: adalimumab
		Pt3: 45/M	Pt3: plaque	Pt3: AZD1222/1	Pt3: 10	Pt3: secukinumab	Pt3: secukinumab + calcip/betam
		Pt4: 61/M	Pt4: plaque	Pt4: BNT162b2/2	Pt4: 12	Pt4: adalimumab	Pt4: ixekizumab
		Pt5: 62/M	Pt5: plaque	Pt5: mRNA-1273/2	Pt5: 8	Pt5: none	Pt5: brodalumab
		Pt6: 47/M	Pt6: guttate	Pt6: BNT162b2/2	Pt6: 9	Pt6: ixekizumab	Pt6: ixekizumab + calcip/betam
		Pt7: 70/F	Pt7: plaque	Pt7: BNT162b2/2	Pt7: 8	Pt7: calcip/betam	Pt7: adalimumab
		Pt8: 39/F	Pt8: plaque	Pt8: AZD1222/2	Pt8: 7	Pt8: guselkumab	Pt8: guselkumab + calcip/betam
		Pt9: 58/M	Pt9: plaque	Pt9: BNT162b2/2	Pt9: 5	Pt9: secukinumab	Pt9: secukinumab + calcip/betam
		Pt10: 55/F	Pt10: plaque	Pt10: AZD1222/2	Pt10: 10	Pt10: nbUVB	Pt10: risankizumab
		Pt11: 59/M	Pt11: plaque	Pt11: BNT162b2/1	Pt11: 14	Pt11: etanercept	Pt11: ixekizumab
Wei et al. [39]	6	Pt1: 76/M	Pt1: plaque	Pt1: mRNA-1273/2	Pt1: 62	Pt1: NR	Pt1: apremilast + nbUVB
		Pt2: 69/M	Pt2: plaque	Pt2: mRNA-1273/2	Pt2: 21	Pt2: NR	Pt2: apremilast + tildrakizumab
		Pt3: 68/F	Pt3: plaque	Pt3: mRNA-1273/2	Pt3: 6	Pt3: NR	Pt3: risankizumab
		Pt4: 67/M	Pt4: plaque	Pt4: mRNA-1273/2	Pt4: 60	Pt4: NR	Pt4: tildrakizumab + TCS
		Pt5: 52/F	Pt5: plaque	Pt5: mRNA-1273/2	Pt5: 7	Pt5: NR	Pt5: risankizumab + TCSt
		Pt6: 27/F	Pt6: plaque	Pt6: BNT162b2/2	Pt6: 90	Pt6: NR	Pt6: TCS
Ständer et al. [40]	4	Pt1: 35/M	Pt1: guttate	Pt1: BNT162b2/2	Pt1: 7	Pt1: NR	Pt1: none
		Pt2: 62/M	Pt2: guttate	Pt2: mRNA-1273/2	Pt2: 10	Pt2: NR	Pt2: ustekinumab
		Pt3: 58/F	Pt3: plaque	Pt3: BNT162b2/2	Pt3: 5	Pt3: NR	Pt3: ixekizumab
		Pt4: 54/M	Pt4: plaque	Pt4: BNT162b2/3	Pt4: 7	Pt4: NR	Pt4: ixekizumab
Ruggiero et al. [50]	4	Pt1: 55/M	Pt1: nail	Pt1: BNT162b2/2	Pt1: 19	Pt1: none	Pt1: methotrexate
		Pt2: 66/M	Pt2: nail	Pt2: mRNA-1273/1	Pt2: 21	Pt2: apremilast	Pt2: ixekizumab
		Pt3: 41/M	Pt3: nail	Pt3: BNT162b2/2	Pt3: 16	Pt3: adalimumab	Pt3: brodalumab
		Pt4: 52/F	Pt4: nail	Pt4: BNT162b2/2	Pt4: 25	Pt4: secukinumab	Pt4: secukinumab + TCS +TK
Durmaz et al. [57]	3	Pt1:64/M	Pt1: plaque	Pt1: BNT162b2/3	Pt1: 42	None	NR
		Pt2: 64/M	Pt2: palmoplantar	Pt2: BNT162b2/2	Pt2: 7		
		Pt3: 25/F	Pt3: pustular	Pt3: BNT162b2/1	Pt3: 3		
Piccolo et al. [58]	2	Pt1: 57/M	Pt1: palmoplantar	Pt1: BNT162b2/1	Pt1: 30	Pt1: TCS	Pt1: acitretin
		Pt2: 63/F	Pt2: palmoplantar	Pt2: BNT162b2/1	Pt2: 30	Pt2: TCS	Pt2: acitretin
Tran et al. [59]	2	Pt1: 30/F	Pt1: Erythrodermic	Pt1: BNT162b2/2	Pt1: 7	Pt1: secukinumab	Pt1: acitretin
		Pt2: 45/F	Pt2: Erythrodermic	Pt2: BNT162b2/2	Pt2: 7	Pt2: calcip/betam	Pt2: NR
Bostan et al. [60]	1	51/M	Plaque	BNT162b2/2	14	NR	NR
Krajewski et al. [61]	1	46/M	Plaque	BNT162b2/2	5	Deucravacitinib	NR
Fang et al. [62]	1	34/F	Plaque	AZD1222/1	7	Ustekinumab + cyclosporine	TCS
Mieczkowska et al. [63]	1	65/M	Plaque	BNT162b2/1	7	Apremilast + calcip/betam	NR
Niebel et al. [64]	1	62/M	Plaque	BNT162b2/2	20	None	Balneophototherapy
Kabbani et al. [65]	1	53/M	Plaque	BNT162b2/1	7	None	Cyclosporine
Burlando et al. [66]	1	56/M	Plaque	BNT162b2/2	16	NR	NR
Pesqué et al. [44]	1	30/F	Plaque	mRNA-1273/1	10	TCS	Calcip/betam
Phuan et al. [67]	1	80/F	Guttate	BNT162b2/3	7	Cyclosporine	Cyclosporine + TCS
Quattrini et al. [68]	1	83/F	Palmoplantar	BNT162b2/2	2	Methotrexate	OCS + methotrexate
Onsun et al. [69]	1	72/M	Pustular	AZD1222/1	4	TCS	Infliximab
Perna et al. [70]	1	NR/M	Pustular	BNT162b2/1	5	None	Infliximab + cyclosporine
Yatsuzuka et al. [71]	1	65/M	Pustular	BNT162b2/2	12	Infliximab	Secukinumab + OCS
Pavia et al. [72]	1	47/F	Pustular	BNT162b2/2	10	Ustekinumab	Risankizumab
Rouai et al. [73]	1	66/M	Pustular	BNT162b2/1	4	None	TCS
Durmus et al. [74]	1	42/M	Erythrodermic	BNT162b2/1	28	Secukinumab	Ixekizumab + OCS
Lopez et al. [75]	1	58/M	Erythrodermic	BNT162b2/2	4	None	TCS
Nia et al. [76]	1	58/M	Erythrodermic	BNT162b2/1	2	None	Cyclosporine + nbUVB + TCS

M: male; F: female; AZD1222: AstraZeneca-Oxford AZD1222; mRNA-1273: Moderna mRNA-1273; BNT162b2: Pfizer mRNABNT162b2; Ad26.COV2.S: Johnson & Johnson; calcip/betam: topical calcipotriol/betamethasone combination; nbUVB: narrow-band UVB; PUVA: psoralen-UVA; TCS: topical corticosteroids; OCS: oral corticosteroids; TK: topical keratolytics; NR: not reported. * Number of days between new-onset psoriasis and vaccination. ** Psoriasis treatment before vaccination. *** Psoriasis treatment following COVID-19 vaccination-related psoriasis flare-up.

## 4. Discussion

Psoriasis is a complex autoimmune disorder characterized by an abnormal immune response that leads to chronic inflammation and the accelerated growth of skin cells [77]. Cytokines play a crucial role in the pathogenesis of psoriasis, serving as key mediators in the inflammatory process [78,79]. In particular, TNFα, IL23, IL17, IL22 and IL6 have been reported to play a central role in the initiation and maintenance of psoriatic inflammation, promoting the recruitment and activation of immune cells, and the production of other cytokines [78,79]. Targeting these cytokines has been a successful approach in the treatment of psoriasis [80,81,82]. Indeed, biologic drugs, such as anti-TNFα, IL17, and IL23, have been developed to specifically block the actions of these cytokines and reduce inflammation in psoriatic skin, revolutionizing the psoriasis treatment scenario [80,81,82].

The COVID-19 pandemic impacted daily clinical practice [83,84]. In particular, several strategies have been adopted to contain the spreading of the infection [85,86]. Among these, vaccination was the main one. However, although the preliminary safety concerns and doubts raised at the beginning of the vaccination campaign related to vaccines’ safety were overcome, several cutaneous adverse events were reported, most of these not being shown in clinical trials [87,88,89]. Fortunately, the majority of these were mild and self-limiting, and did not require medical attention [90,91]. In addition, the vaccination campaign was also limited by several personal burdens (a fear of vaccination and its side effects, stress from needing a vaccination to travel or work, etc.) [84,92,93].

As regards psoriasis, several cases of exacerbation or a new onset of the disease were reported. However, comprehensive manuscripts collecting all these data in order to offer a wide perspective are scant. In this context, we performed a review with the purpose of showing a wide analysis of COVID-19 vaccination and psoriasis development/exacerbation and pointing out possible pathogenetic mechanisms. It is important to note that the information provided is based on the current understanding of these topics and may evolve as further research becomes available. Globally, in total, 49 studies involving 134 patients developing new-onset psoriasis (*n* = 27, 20.1%) or experiencing a psoriasis exacerbation (*n* = 107, 79.9%) were collected. In both cases, mRNABNT162b2 was the commonest vaccine associated and plaque psoriasis was the commonest clinical phenotype, while a significant gender predominance was not reported and cutaneous reactions were reported following each dose of a vaccine. In our opinion, mRNABNT162b2 was the commonest vaccine related to cutaneous reactions since it was the most commonly used during the vaccination campaign, and plaque psoriasis is the commonest clinical phenotype according to psoriasis phenotype epidemiology. Moreover, all of the cases have been successfully treated with topical or systemic medications, including biologics. In particular, a difference between patients starting or switching to biologic treatment for psoriasis has been found between new-onset and flare-up groups (11.1% vs. 36.4%). In our opinion, the increased awareness of psoriatic disease in patients already suffering from the disease has reduced the number of consultations for mild exacerbations. Indeed, patients affected by psoriasis are more used to self-medication with topical drugs, reducing the need for medical advice in the case of mild forms of the disease. This may explain the difference between the predominance of moderate-to-severe forms of disease in patients who developed a psoriasis flare-up and those with mild forms in de novo cases where the use of topical treatments for the management was predominant (33.3% vs. 21.5%). Unfortunately, ongoing treatment prior to COVID-19 vaccination was often not reported, which prevented the results from being analyzed to reveal whether or not some psoriatic treatments may increase the risk of disease exacerbation.

Of interest is that 19 (17.8%) patients who developed psoriasis exacerbation were under biologic treatment for psoriasis at the moment of the flare-up. Reviewing the current literature, being on biologic drugs at the moment of vaccination seems to reduce the risk of psoriasis worsening [30,31,94,95], but clinical studies comparing patients undergoing biologics and patients receiving other medications and/or a placebo at the moment of vaccination are absent, not allowing a confirmation of these data. Certainly, biologic drugs were also shown to be safe and effective during the pandemic period.

In addition, the number of days between new-onset psoriasis or the exacerbation of psoriasis and vaccination is not reported in most of the studies. Moreover, the onset or exacerbation of nail psoriasis should also be discussed. Indeed, there are few cases reporting the onset or worsening of this form of disease and the time between vaccination and reporting is too short to limit this condition to the COVID-19 vaccine.

Thus, while there have been anecdotal reports of new-onset psoriasis or psoriasis exacerbation following COVID-19 vaccination, it is essential to evaluate these cases in the context of existing scientific knowledge. Firstly, there is currently no direct evidence linking COVID-19 vaccination to the development of psoriasis. Vaccines, including COVID-19 vaccines, work by stimulating the immune system to produce a protective response against the virus [96,97,98]. The mechanisms involved in vaccine-induced immune responses are different from those implicated in psoriasis pathogenesis [96,97,98]. It is unlikely that COVID-19 vaccination would directly trigger the development of psoriasis in individuals without a pre-existing predisposition [96,97,98]. However, it is important to consider the potential of immune system activation or modulation following vaccination [96,97,98]. In some cases, vaccines can induce immune responses that may lead to transient inflammation or immune system activation [96,97,98]. This immune activation may theoretically contribute to the exacerbation of pre-existing psoriasis in individuals already diagnosed with the condition [96,97,98]. In addition, the induction of neutralizing antibodies and T-cell responses via vaccination may lead to an increasement and production of TNFα and Interferon (IFN) γ [96,97,98]. Similarly, vaccination can activate plasmacytoid and dermal myeloid dendritic cells which may be a trigger for the psoriasis cascade [96,97,98]. Finally, vaccinations might induce the production of IL6, which may be a trigger for Th17 cells to produce IL22, which itself stimulates keratinocyte proliferation [96,97,98].

Notably, cases of de novo psoriasis or an exacerbation of psoriasis have been reported following both mRNA and viral vector-based vaccines, suggesting that the onset or the worsening of the disease is not related to the mechanism of action of the vaccines but to the vaccination itself.

Furthermore, it is crucial to differentiate between coincidence and causation when assessing the relationship between COVID-19 vaccination and new-onset psoriasis. Psoriasis is a relatively common skin condition, and it is possible for new cases to emerge coincidentally after vaccination, without a direct causal relationship. Robust epidemiological studies and careful evaluation of individual cases are needed to determine any potential association between COVID-19 vaccination and the development of psoriasis. Moreover, it is mandatory to emphasize the overall benefit–risk profile of COVID-19 vaccination. Indeed, COVID-19 is a severe and potentially life-threatening illness, and the benefits of vaccination in preventing infection, reducing severe disease, and limiting the spread of the virus far outweigh the potential risks.

## 5. Strengths and Limitations

The number of investigated studies and the literature review using the PRISMA methods are the main strengths of our manuscript. The absence of clinical studies and consistent data such as data from registries are the main limitations. Moreover, we hypothesize that cases of psoriasis exacerbations or de novo disease developed following vaccination were underestimated, since not all patients seek medical advice due to the limited severity of the disease, tending to self-medicate (particularly in patients affected by psoriasis who are more accustomed to self-medication with topical drugs, reducing the need for medical advice in the case of mild forms of the disease), and many reactions have not been reported. Finally, psoriatic arthritis was not considered in our manuscript.

## 6. Conclusions

To sum up, the COVID-19 vaccination campaign was a success. Although cases of de novo psoriasis or disease worsening have been reported following vaccination, all of the cases have been successfully treated (mainly with topicals in de novo cases and systemic treatments in psoriasis flare-ups), while the overall benefit–risk profile of COVID-19 vaccination do not justify the vaccine hesitancy due to the risk of psoriasis being developed or worsening. Globally, plaque psoriasis was the most common clinical phenotype both in terms of de novo psoriasis and the exacerbation of psoriasis. Moreover, more severe forms of the disease have been reported in patients with a history of psoriasis compared to the new onset cases where mild forms were predominant. This difference may be explained by psoriatic patients’ ability to self-medicate with topical drugs for mild forms of the disease, reducing the need for medical advice for moderate-to-severe conditions. Certainly, further studies are needed to identify possible pathogenetic mechanisms in order to identify “at-risk” patients. Finally, vaccination should not be discouraged.

## Data Availability

Data are reported in the current study.
